# Pharmacokinetics and Biologic Activity of Apixaban in Healthy Dogs

**DOI:** 10.3389/fvets.2021.702821

**Published:** 2021-07-05

**Authors:** Noelle D. Herrera, Ingvild Birschmann, Monika Wolny, Mark G. Papich, Marjory B. Brooks, Robert Goggs

**Affiliations:** ^1^Department of Clinical Sciences, Cornell University College of Veterinary Medicine, Ithaca, NY, United States; ^2^Institute for Laboratory and Transfusion Medicine, Heart and Diabetes Center North Rhine-Westphalia, Bad Oeynhausen, Germany; ^3^Department of Molecular Biomedical Sciences, North Carolina State College of Veterinary Medicine, Raleigh, NC, United States; ^4^Department of Population Medicine and Diagnostic Sciences, Cornell University College of Veterinary Medicine, Ithaca, NY, United States

**Keywords:** antithrombotic, anti-Xa activity, direct oral anticoagulant, thrombosis, thromboelastography

## Abstract

Thrombosis is common in critically ill dogs and causes considerable morbidity and mortality. The direct factor Xa inhibitor apixaban is safe, efficacious, and convenient in humans. This study aimed to determine the pharmacokinetics (PK), bioactivity, protein binding, and bioavailability of apixaban following intravenous (IV) and oral (PO) administration to healthy dogs. Six healthy, adult, mixed-breed dogs were administered apixaban 0.18 mg/kg IV and then following a minimum 2-week washout period administered apixaban 0.2 mg/kg PO. Dogs were monitored using an apixaban-calibrated anti-Xa bioassay, prothrombin time (PT) and activated partial thromboplastin time (aPTT) and tissue-factor thromboelastography (TF-TEG). Plasma apixaban concentrations were measured using liquid chromatography-tandem mass spectrometry. Concentration-time plots were constructed, and PK modeling performed using compartmental methods. Administration of IV and PO apixaban was well-tolerated. Following IV administration, mean half-life was 4.1 h, and volume of distribution was 177 ml/kg. Apixaban was highly protein bound (98.6%). Apixaban concentrations and anti-Xa activity were highly correlated (R^2^ 0.994, *P* < 0.0001). Intravenous apixaban significantly prolonged PT at time points up to 1 h, and aPTT at time points up to 0.25 h post-administration. Coagulation times were positively correlated with apixaban concentrations (PT R^2^ 0.599, *P* < 0.0001; aPTT R^2^ 0.430, *P* < 0.0001) and TF-TEG R-time was significantly prolonged 0.25 h post-administration. Following oral administration, mean bioavailability was 28.4%, lag time was 2 h, time to C_max_ was 5 h and the apparent elimination half-life was 3.1 h. Oral apixaban significantly prolonged PT at 4, 6, and 8 h but aPTT and TF-TEG were not consistently affected by oral apixaban. Apixaban concentrations are best monitored using anti-Xa activity. Future studies should determine PK and bioactivity of other doses using commercial tablets and following multidose administration and establish safe, effective dosing ranges in sick dogs.

## Introduction

Thrombosis is commonly encountered in critically ill dogs (1, 2) as a severe complication of acquired conditions including immune-mediated hemolytic anemia, pancreatitis, and protein-losing nephropathy (3–5). Thrombosis causes considerable morbidity and mortality due to direct obstruction of blood flow resulting in end-organ injury and through embolization and feedback amplification of the inflammatory response (6–8). As such, there is a critical need for efficacious, safe, and cost-effective anticoagulants for thromboprophylaxis and treatment of existing thrombi. A new class of direct oral anticoagulants (DOACs) includes the factor Xa inhibitors apixaban, rivaroxaban, and edoxaban (9–11). These drugs were developed as orally absorbed anticoagulants with better safety profiles than warfarin (12). Warfarin has a narrow therapeutic index and is subject to numerous drug and diet interactions and thus requires frequent monitoring to prevent hemorrhagic complications or recurrent thrombosis. Data on warfarin in dogs suggest its effects vary widely between animals (13) and its use is no longer recommended due to difficulties in monitoring and risk for hemorrhage (14). While heparins represent an alternative class of anticoagulants, many owners are reluctant to give subcutaneous injections (15) and these drugs still require monitoring and dose adjustments to maximize safety and efficacy (16).

The DOACs hold promise to revolutionize anticoagulant therapy in veterinary medicine with the same advantages of safety, efficacy, and convenience that have improved thromboprophylaxis in humans. Accordingly, recent consensus guidelines formulated by veterinary criticalists recommend further investigation of the direct Xa inhibitors (14). The *in vitro* pharmacology and the pharmacokinetics and pharmacodynamics of rivaroxaban have been studied in dogs and cats (17, 18), and it appears to be effective in dogs (19, 20). Apixaban, another factor Xa inhibitory drug, was approved by the FDA in 2012 for prevention of stroke or systemic embolism in humans with atrial fibrillation (11) without individual patient monitoring. Evidence from human medicine suggests that within the DOACs, individual drugs have distinct efficacy and bleeding risk (21). Meta-analyses suggest that apixaban could be more effective than rivaroxaban (22), and carry lower risk of major bleeding than other DOACs (23, 24). Further investigation of apixaban in veterinary medicine is therefore warranted. Studies of apixaban have been conducted in horses (25) and in cats (26). Evaluation of apixaban in dogs is limited to a single clinical case report (27) and preclinical drug toxicity studies (28–30). The objectives of this study were to determine the pharmacokinetics and biologic activity of apixaban following intravenous (IV) and oral (PO) administration to healthy dogs, and to measure the degree of protein binding and assess the oral bioavailability of the available drug formulation. It was hypothesized that apixaban has reproducible pharmacokinetics in dogs, is highly protein bound (>90%), has good oral bioavailability (>80%) and that plasma drug concentrations are positively correlated with biologic activity as measured by factor Xa inhibitory (anti-Xa) bioassay.

## Materials and Methods

### Animals

Six University-owned, healthy mixed-breed dogs (four female, two male) aged 4.1–6.4 years old and weighing between 10.5–17.2 kg were used for this study. Mixed-breed dogs were used because prior publications have suggested beagles might respond differently to anticoagulants than other breeds (31, 32). Additionally, drug metabolism might be different in beagles compared with mixed-breed dogs (33, 34). Dogs had food withheld for 8 h prior to drug administration to reduce the risk of aspiration following sedation and to minimize any interference with gastrointestinal drug absorption. The study was approved by the Institutional Animal Care and Use Committee (IACUC) at Cornell University College of Veterinary Medicine (Protocol #2019-0099). All dogs were housed at Cornell University and cared for by licensed veterinarians and technicians according to established protocols. All dogs were permanently adopted into private homes after study completion.

### Drug Preparation and Dosing

A dose of 0.2 mg/kg apixaban was chosen based on prior studies in cats and horses (25, 26). To achieve uniform dosing, custom IV (2.5 mg/ml) and oral capsule formulations were created. The protocol for generation of the IV formulation was as previously published (25), and all procedures were performed in a laminar flow hood under strict aseptic conditions. To generate an IV apixaban formulation, commercial tablets (Eliquis, Bristol-Myers Squibb, New York, NY) were triturated, sieved using a 100 μm filter and then dissolved in a vehicle consisting of 10% v/v N,N-dimethylacetamide, 17% v/v propylene glycol, 17% v/v dimethyl sulfoxide (all from Millipore-Sigma, St. Louis, MO), and 56% v/v deionized water for injection (Hospira, Lake Forest, IL). After vortexing, the formulation was aseptically filtered (Corning 0.22 μm cellulose acetate filter, Millipore-Sigma) then drawn into 10 ml syringes (BD, Franklin Lakes, NJ). The IV formulation was prepared 1 h before administration and stored in the dark at 20-25°C. For the oral capsule formulations, apixaban tablets (Eliquis 5 mg, Bristol Myers Squibb) were crushed to a fine powder and triturated to obtain a uniform particle size. The powder was then mixed with dextrose powder, anhydrous, USP (PCCA, Houston, TX) to create custom capsules (size #3 gelatin locking capsules, PCCA) each containing 2, 2.5, or 3 mg of apixaban and stored at 20-25°C prior to administration. These capsules had a 180d expiration date. All dogs received 0.4 ml/kg of the 0.5 mg/ml intravenous solution aiming to provide 0.2 mg/kg apixaban by slow IV bolus injection over 2 min. All injection durations were identical. Separately, all dogs received bodyweight appropriate capsule size to provide ~0.2 mg/kg PO once. Capsules were administered without food and witnessed to be ingested. A minimum washout period of 2 weeks elapsed between initial PO administration and subsequent IV administration. The maximum duration of capsule storage prior to administration was 76 days.

### Sample Collection

On the days of drug administration, 5 Fr × 25 cm trilumen wire-guided catheters (Mila International, Erlanger, KY) were aseptically placed in external jugular veins by modified-Seldinger technique to facilitate atraumatic, repeat blood sample collection. Dogs were sedated with IV butorphanol 0.2 mg/kg and dexmedetomidine 5 μg/kg (Zoetis, Parsippany, NJ) to facilitate catheter placement. Jugular catheters were secured using 3-0 nylon sutures, protected with sterile bandages, and flushed with 0.9% saline (no heparin was used). Dogs were allowed to recover fully from sedation prior to drug administration. For IV apixaban administration, one catheter lumen was dedicated to drug administration and separate catheter lumens used for sample collection to prevent sample contamination. Blood samples were collected using a 3-syringe technique, with replacement, into 2.7 ml evacuated tubes (BD) containing buffered 3.2% citrate at baseline, then 5, 15, and 30 min and 1, 2, 4, 8, 12, and 24 h after drug administration. Catheters were flushed with 0.9% saline, without heparin. Samples were also drawn into K_2_-EDTA and no-additive tubes at baseline and 24 h post-administration for complete blood counts and serum biochemistry profiles. Dogs were closely monitored for adverse reactions throughout the sample collection period. Jugular catheters were removed after the 12 h time point and samples collected *via* direct venipuncture at the 24 h timepoint. After each sample collection, one citrate anticoagulated whole blood sample was analyzed using thromboelastography (TEG), while the second citrate sample was centrifuged (1,370 g, 10 min, 20°C), the plasma separated, split into two aliquots and then frozen at −80°C pending batch analyses. For oral drug administration, all procedures were as described above, except for that apixaban was administered orally (and witnessed to have been ingested), and samples were drawn at baseline, then 1, 2, 4, 6, 8, 12, 24, and 30 h post-drug administration.

### Coagulation Testing and TEG

Coagulation assays were performed on platelet-poor, citrated plasma samples by a reference laboratory (Comparative Coagulation Laboratory, Animal Health Diagnostic Center, Ithaca, NY). Apixaban anti-Xa activity (aXa) was measured using an automated coagulation analyzer (STA Compact, Diagnostica Stago, Parsipanny, NJ) with the manufacturer's reagents, calibrators, and controls according to the manufacturer's recommendations. The assay is configured with a bovine activated Factor X reagent that is added in excess to the test plasma and a chromogenic substrate of Factor Xa (STA Liquid anti-Xa, Diagnostica Stago). Residual, uninhibited Factor Xa cleaves the chromogenic substrate such that the inverse of the color change in the reaction mixture is proportional to the drug concentration in the test plasma. Results are expressed as ng/ml of aXa activity, based on a calibration standard containing known apixaban concentrations in human plasma (STA Apixaban Calibrator, Diagnostica Stago). Assay controls, consisting of human plasma spiked with apixaban (STA Apixaban Controls, Diagnostica Stago) were analyzed before each batch of test samples to confirm assay performance. The apixaban levels in calibrators and controls are defined by the manufacturer from an internal reference assayed by HPLC-MS. The assay's linearity range extends from 23 to 500 ng/ml apixaban, with values falling outside this range extrapolated from the standard curve. A mechanical endpoint method (STA Compact, Diagnostica Stago) and human coagulation reagents were used to measure prothrombin time (Thromboplastin LI, Helena Diagnostics, Beaumont, TX) and activated partial thromboplastin time (Dade Actin FS, Dade Behring, Newark, DE). The local reference interval for the prothrombin time was 11.5–15.5 s and for the activated partial thromboplastin time 8.5–15.5 s. Tissue-factor (1:50,000 final dilution) activated TEG was performed using standard equipment (TEG-5000, Haemonetics, Braintree, MA) as previously reported (35), with assays conducted per established guidelines (36), with a modification to eliminate the rest time to facilitate rapid sample processing. Thromboelastography assays were conducted by experienced operators using four TEG channels and tracings terminated after maximum amplitude was established. Four values were extracted from TEG tracings: reaction time (R-time), clot formation time (K-time), clot formation angle (alpha), and maximum amplitude (MA).

### Measurement of Apixaban Concentrations

For quantitation of plasma apixaban concentrations, samples were shipped by courier on dry ice to the Institute for Laboratory and Transfusion Medicine, Heart and Diabetes Center NRW, Bad Oeynhausen, Germany. Apixaban was quantitated as previously described (37) using automated online solid-phase extraction followed by LC/MS performed on protein-free samples and hence is species independent. In brief, proteins in the sample were initially precipitated by combining 100 μl of plasma sample with 200 μl of 0.1 M ZnSO_4_ solution and 700 μl of isotope standard stock solution, mixing in a vortex device for 5 s followed by centrifugation (14,000 g, 5 min, RT). The supernatant was added to glass autosampler vials and an aliquot injected into the liquid chromatography 2D UPLC system (Waters Acquity UPLC H-class with 2D Technology System). Sample extraction was performed using a 2.1 × 30 mm reverse phase cartridge (column 1, Waters, XBridge C8, 10 μm) followed by an analytical separation on a 2.1 × 50 mm reverse phase column (column 2, Waters, Acquity UPLC BEH C18, 1.7 μm) and an elution with a linear gradient using water containing 0.1% formic acid and 2 mmol/L ammonium acetate and methanol containing 0.1% formic acid and 2 mmol/L ammonium acetate. The mass spectrometer (Xevo TQ-S, Waters) was used in electrospray positive ionization mode with the transition monitored for apixaban at m/z 460.3 → 443.1 and 460.3 → 199.1 as well as the internal standard [^13^C,^2^H_7_]-apixaban at m/z 468.3 → 451.1 and 468.3 → 199.1. Plasma concentrations of apixaban were calculated by use of linear regression analysis and extrapolation from a calibration curve generated by use of blank dog plasma fortified with increasing concentrations of apixaban. A 10-point calibration curve was generated with concentrations ranging from 1 to 1,000 ng/ml, with three quality control samples at each of three concentrations (low, 2.5 ng/ml; medium, 50 ng/ml; high, 500 ng/ml). The limit of detection was 0.2 ng/ml and the lower limit of quantitation was 0.6 ng/ml, but because values <7 ng/ml are not clinically relevant, the laboratory does not report values below 7 ng/ml.

### Pharmacokinetic and Pharmacodynamic Modeling

Apixaban plasma concentrations were plotted on semilogarithmic graphs for visual assessment of the best model for pharmacokinetic analysis. Analyses of plots and PK modeling were performed using compartmental methods in standard software (Phoenix, WinNonlin, Certara, St. Louis, MO). One-, 2- and 3-compartment models were fit to the plasma concentration data. After examination of the diagnostic plots, residual plots, and Akaike information criterion (38), a one-compartment model was selected for the IV dose data according to the equation:

$$\begin{array}{l} C=\frac{D}{V}\;\times e^{\left(-K10\;\cdot T\right)} \end{array}$$

Where C is the plasma drug concentration, V is the volume of distribution, D is the dose, K10 is the first order elimination rate constant, and T is time. Secondary parameters included area-under-the curve (AUC), systemic clearance (CL), and half-life. For oral administration, the model that best fit the data involved first-order input, with a lag time (T_Lag_) to allow for capsule dissolution and stomach emptying, followed by first-order elimination according the to the equation:

$$\begin{array}{l} C=\frac{D\cdot K01}{V\left(K01-K10\right)}\times \left[\left(e^{-K10\cdot \left(T-T_{Lag}\right)}\right)\;-\left(e^{-K01\cdot \left(T-T_{Lag}\right)}\right)\right] \end{array}$$

Where C is the plasma drug concentration, V is the volume of distribution, K10 is the first order elimination rate constant, K01 is the absorption rate constant, and T is time. Secondary parameters included area-under-the curve (AUC), systemic clearance (CL), and half-lives. Absolute bioavailability of apixaban for each dog was estimated from the plasma area under the curve data after IV and PO administration as follows: F = (AUC_PO_/AUC_IV_) × (Dose_IV_/Dose_PO_).

Pharmacodynamic efficacy was modeled using estimates of the half-maximal effective concentration (EC_50_) and the efficacy (E_*max*_) of apixaban based on anti-Xa activity, as follows:

$$\begin{array}{l} E=\frac{\left(E_{\max}\;\times \;C\right)}{\left(C+EC_{50}\right)\;} \end{array}$$

### Protein Binding

The extent of protein binding by apixaban was measured by *ex vivo* ultrafiltration using a cellulose centrifugation device followed by LC/MS analysis of the protein-free ultrafiltrate. For each dog a sample of anticoagulated platelet-poor plasma collected at baseline was centrifuged (16,000 g, 5 min, 4°C) and the supernatant filtered with a 0.22 μM syringe filter. Plasma supernatants were spiked with apixaban at 500 ng/ml using the IV apixaban formulation and divided into two aliquots. One aliquot was centrifuged (16,000 g, 30 min, 4°C) in a molecular exclusion device (Amicon Ultra 30k filter, Millipore-Sigma) to remove the major plasma proteins. The spiked plasma and the ultrafiltrate were then analyzed for apixaban by LC/MS as above. Protein binding (%) was determined as: [Total concentration] – [Protein – Unbound concentration]/Total concentration ×100.

### Data Analysis

Pairwise comparisons between continuous variables were conducted with Wilcoxon matched-pairs signed rank test, while comparisons of more than two groups were conducted using the Friedman test with Dunn's *post-hoc* adjustment. Specifically, the Wilcoxon matched-pairs signed rank tests were used to compare the complete blood count and serum chemistry values between baseline and the +24 h time point. Comparisons of pharmacodynamic variables between baseline values and those at discrete sampling intervals were performed using the Friedman test with Dunn's *post-hoc* correction for multiple comparisons. Calculation of Pearson correlation coefficients and linear least-squares regression analyses were used to compare plasma apixaban concentrations and coagulation and TEG parameters. Non-linear curve fitting (exponential plateau) was used to assess the association between plasma apixaban concentrations as quantitated by LC/MS and the aXa activity. Alpha was set at 0.05. Statistical analyses were performed using commercial software (Prism 9, GraphPad, La Jolla, CA).

## Results

### Safety Studies

Administration of apixaban both IV and PO was well-tolerated in all dogs. No animals were withdrawn from the study and no adverse reactions, including signs of abnormal bleeding or bruising, were observed during the study period. Following IV administration, statistically significant reductions in chloride and GGT were observed between baseline and 24 h post-injection (Supplementary Figure 1 and Table 1). No changes in CBC parameters were noted following IV administration. Following PO administration, no changes in biochemical parameters were noted between baseline and 30 h post-dose. A significant reduction in mean corpuscular hemoglobin concentration was between baseline and 30 h following administration (Supplementary Figure 1). All other values were unchanged from baseline.

### Intravenous Apixaban

**Figure 1:**
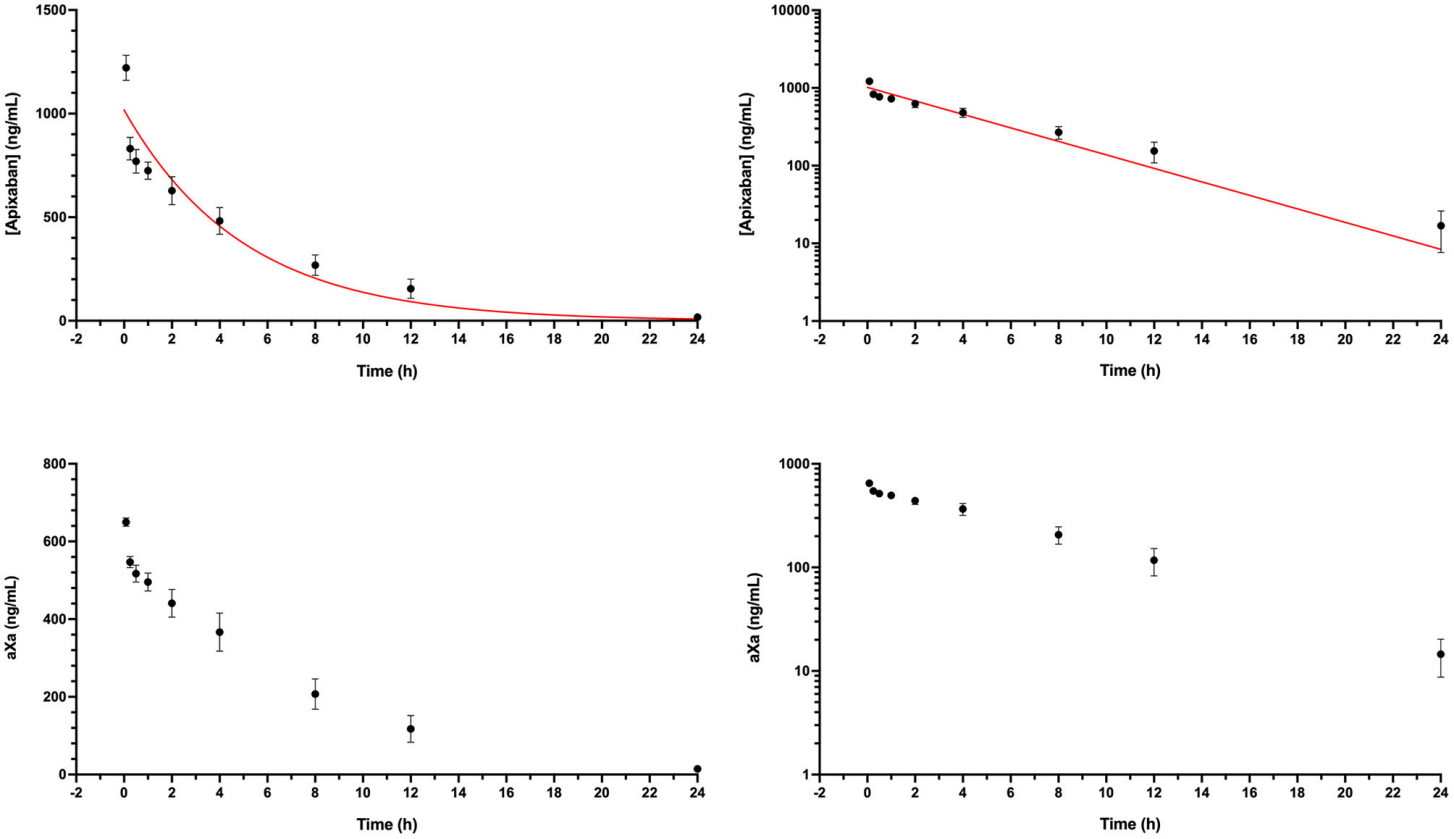
Plasma apixaban concentrations as quantitated by LC/MS and corresponding anti-Xa activity values (aXa) over time following intravenous administration of apixaban (0.18 mg/kg) to 6 healthy mixed-breed dogs. Data presented on the left panels represent mean ± standard deviation values plotted on linear axes, while data on the right panels are displayed on semi-log plots (log_10_ concentration and aXa). Where the standard deviation values were smaller than the width of the dot, the error bars are not displayed. Red lines represent the fit line from the one-compartment pharmacokinetic model (see equation in section Materials and Methods) plotted using mean data (Table 1).

**Figure 2:**
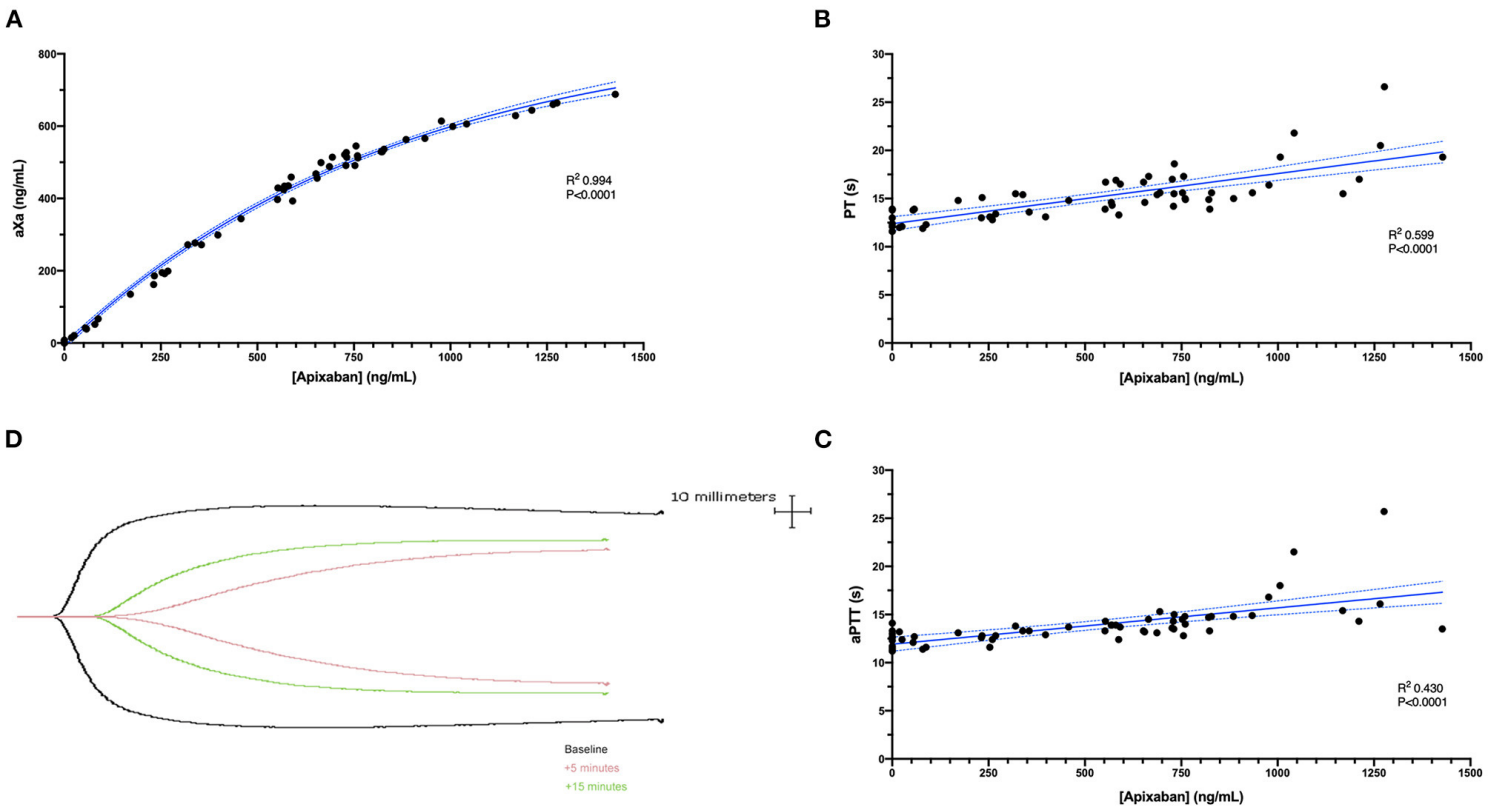
(**A**) A scatterplot demonstrating the non-linear correlation between the plasma concentration of apixaban (abscissa) and the anti-Xa activity (aXa) (ordinate) following intravenous administration of apixaban (0.2 mg/kg). The correlation was highly positive (R^2^ 0.994, *P* < 0.0001). The solid blue line represents the non-linear regression line (exponential plateau), while the dotted blue lines represent the 95% confidence intervals of this line. (**B,C**) Scatterplots demonstrating the correlations between apixaban plasma concentrations (abscissa) and the prothrombin time (PT) or activated partial thromboplastin time (aPTT) (ordinate). These parameters were both positively correlated with the plasma apixaban concentrations (PT R^2^ 0.599, *P* < 0.0001; aPTT R^2^ 0.430, *P* < 0.0001). Solid blue lines represent the Pearson least-squares linear regression lines, while the dotted blue lines represent the corresponding 95% confidence intervals. (**D**) Representative TEG tracings (black, baseline; pink, 5 min; green 15 min) from a single dog following intravenous apixaban administration.

Dogs were administered apixaban by the IV route on three separate occasions (two dogs per day). The final concentrations of the three intravenous preparations varied, such that dogs received a mean of 0.18 ± 0.02 mg/kg. The measured mean plasma concentration following intravenous injection was 1,221 ng/ml, which produced a mean aXa activity of 650 ng/ml. Plasma mean and individual dog apixaban concentration and bioactivity levels were quantifiable throughout the 24-h sampling period (Figure 1). The mean residual concentration was 17 ng/ml at 24 h, mean half-life was 4.1 ± 1.4 h, and the mean volume of distribution was 177 ± 28.7 ml/kg. Pharmacokinetic parameters are summarized in Table 1. Apixaban aXa activity remained above the lower bound of 150 ng/ml, the observed value after 12 h for humans receiving a single intravenous apixaban injection (9, 39). The plasma concentrations of apixaban and the corresponding aXa activity were highly correlated, R^2^ 0.994, *P* < 0.0001 (Figure 2A). Estimated efficacy parameters are summarized in Table 2. All of the deproteinated samples had an apixaban concentration <7 ng/ml and hence an absolute value was not reported. The mean protein binding of apixaban was 98.6 ± 0.1%, using 7 ng/ml for the deproteinated samples.

**Table 1:** Summary of pharmacokinetic parameters following intravenous administration of apixaban, mean dose 0.18 mg/kg.

**Parameter**	**Units**	**Dog 1**	**Dog 2**	**Dog 3**	**Dog 4**	**Dog 5**	**Dog 6**	**Mean**	**SD**	**Geo. Mean**	**CV%**
AUC	h*ng/ml	5,726.9	4,802.6	7,639.4	8,141.5	7,330.9	2,516.3	6,026.3	2,130.0	5,621.4	35.3
CL	ml/h/kg	34.4	41.0	23.4	22.0	20.9	60.8	33.8	15.5	31.2	45.8
C_0_	ng/ml	1,208.2	853.9	1,108.0	958.3	1,005.0	913.4	1,007.8	130.6	1,000.9	13.0
K10	1/h	0.2	0.2	0.2	0.1	0.1	0.4	0.2	0.1	0.2	46.9
K10 T½	h	3.3	3.9	4.8	5.9	5.1	1.9	4.1	1.4	3.9	34.3
MRT	h	4.7	5.6	6.9	8.5	7.3	2.8	6.0	2.1	5.6	34.3
V_d_	ml/kg	163.1	230.7	161.6	186.8	152.2	167.5	177.0	28.7	175.2	16.2

*AUC, area-under-the-curve; CL, systemic clearance; C_0_, concentration at time zero; K10, first order elimination rate, and respective half-life (T½); MRT, mean residence time; V_d_, volume of distribution*.

**Table 2:** Summary of pharmacodynamic efficacy parameters for apixaban anti-Xa activity following intravenous administration, mean dose 0.18 mg/kg.

**Parameter**	**Estimate**	**SE**	**CV (%)**
E_*max*_	1400.5 ng/ml	66.5	4.7
EC_50_	1344.7 ng/ml	101.6	7.6

*CV, coefficient of variation; EC_50_, concentration of a drug that gives half-maximal response' E_max_, concentration giving maximal efficacy; SE standard error*.

**Figure 3:**
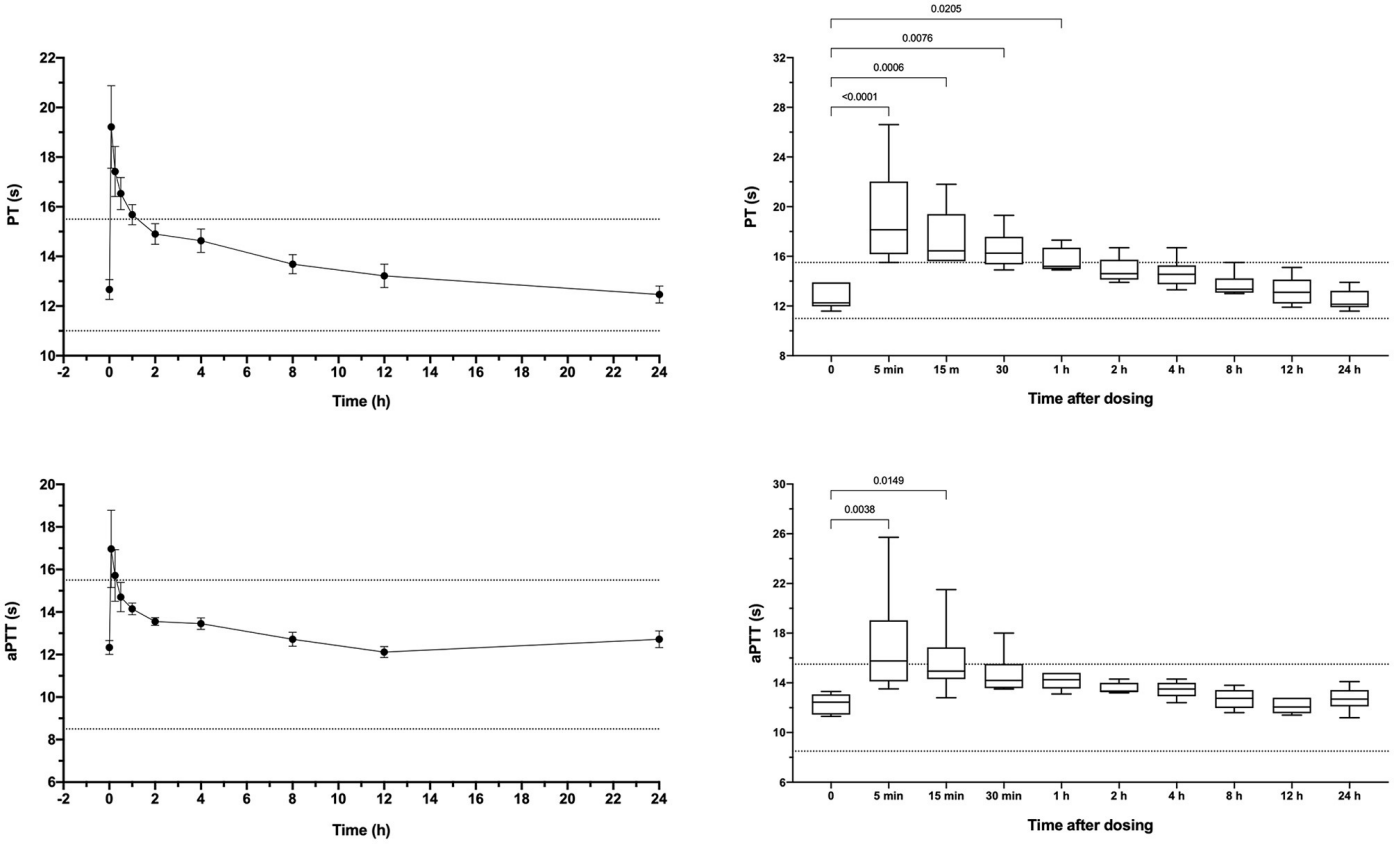
Dotplots and box-whisker plots representing the prothrombin time (PT) and activated partial thromboplastin time (aPTT) values following intravenous administration of apixaban (0.2 mg/kg). Data presented on the left panels represent mean ± standard deviation values, while data on the right panels represent median (middle line), 25–75% percentile (box) and min-max (whiskers) for each time point. Dotted horizontal lines represent the upper and lower bounds of the laboratory reference interval. For the box-whisker plots, comparisons between baseline and subsequent time points were conducted using the Friedman test with Dunn's *post-hoc* correction for multiple comparisons. Only those comparisons that attained *P* < 0.05 are displayed.

**Figure 4:**
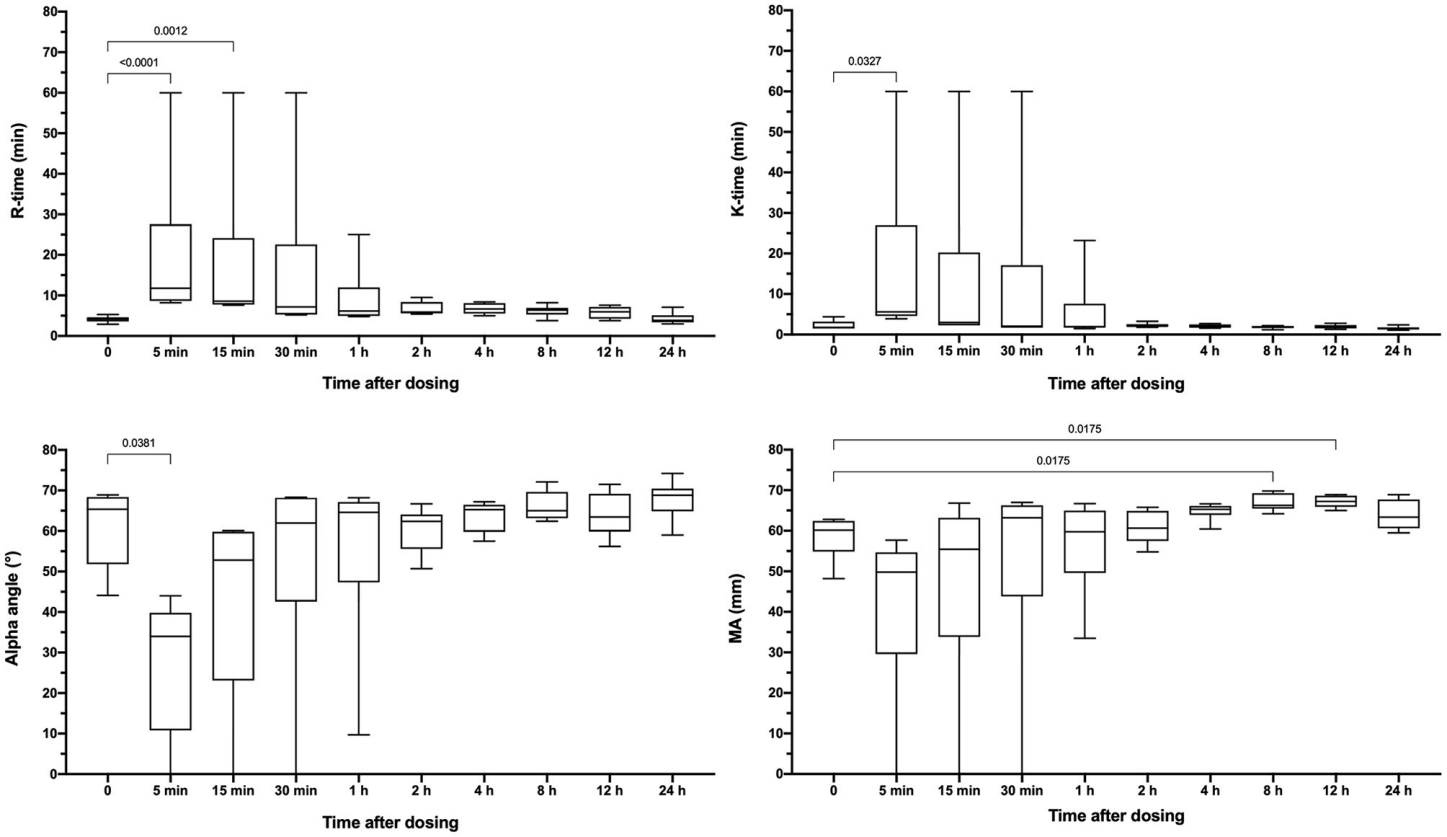
Box-whisker plots representing the reaction time (R-time), clot formation time (K-time), clot formation angle (alpha) and maximum amplitude (MA) from tissue-factor activated thromboelastography tracings. Partial thromboplastin time (aPTT) values following oral administration of apixaban (0.2 mg/kg). Data are presented as median (middle line), 25–75% percentile (box) and min-max (whiskers) for each time point. Comparisons between baseline and subsequent time points were conducted using the Friedman test with Dunn's *post-hoc* correction for multiple comparisons. Only those comparisons that attained *P* < 0.05 are displayed.

Apixaban administered IV significantly prolonged the PT at 5, 15, 30, and 60 min compared to baseline values (Figure 3). Mean values for the PT were also greater than the upper bound of the reference interval at these time points. Intravenous apixaban also significantly prolonged the aPTT at 5- and 15-min post-injection, when the mean values were similarly greater than the upper bound of the reference interval. Values for the PT and aPTT were significantly positively correlated with apixaban plasma concentrations with R^2^ 0.599, *P* < 0.0001 for PT and R^2^ 0.430, *P* < 0.0001 for aPTT (Figures 2B,C). Tissue-factor-activated TEG tracings also demonstrated the effects of intravenous apixaban. Values for R-time, K-time, and alpha angle were all significantly different from baseline at 5 min, with R-time also significantly prolonged at 15 min (Figure 4, with representative traces in panel D, Figure 2). Values derived from TF-activated TEG traces were significantly correlated with apixaban plasma concentrations. Apixaban plasma concentrations were positively correlated with R-time R^2^ 0.286, *P* < 0.0001 and K-time R^2^ 0.122, *P* = 0.0078 and negatively correlated with alpha angle R^2^ 0.273, *P* < 0.0001 and with MA R^2^ 0.187, P = 0.0008 (Supplementary Figure 2).

### Oral Apixaban

**Figure 5:**
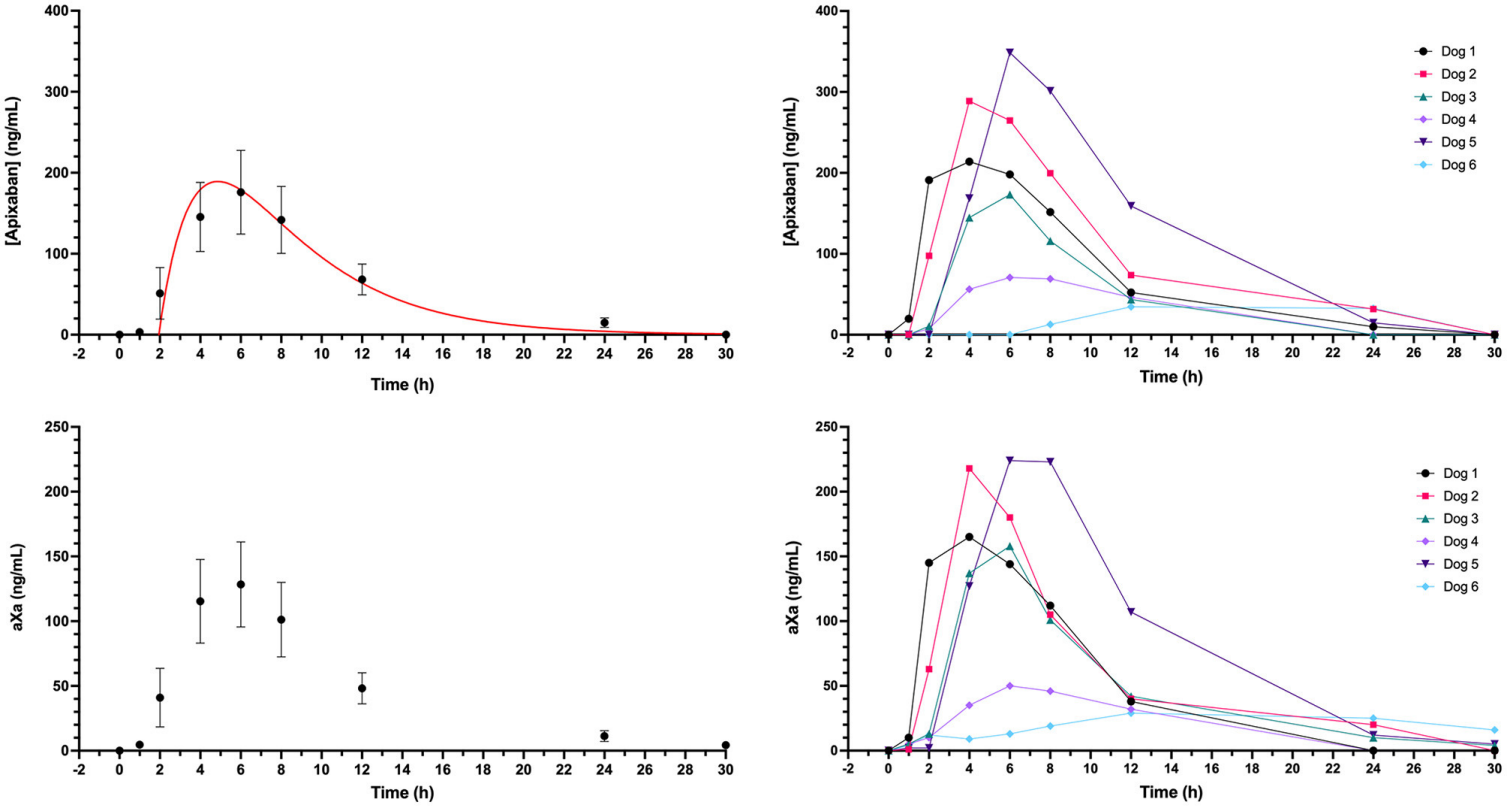
Plasma concentrations and corresponding anti-Xa activity values (aXa) over time following oral administration of apixaban (0.2 mg/kg) to six healthy mixed-breed dogs. Data presented on the left panels represent mean ± standard deviation values, while data on the right panels are from each dog individually. Where the standard deviation values were smaller than the width of the dot, the error bars are not displayed. Red lines represent the fit line from the one-compartment pharmacokinetic model (see equation in section Materials and Methods) plotted using mean data (Table 3).

**Figure 6:**
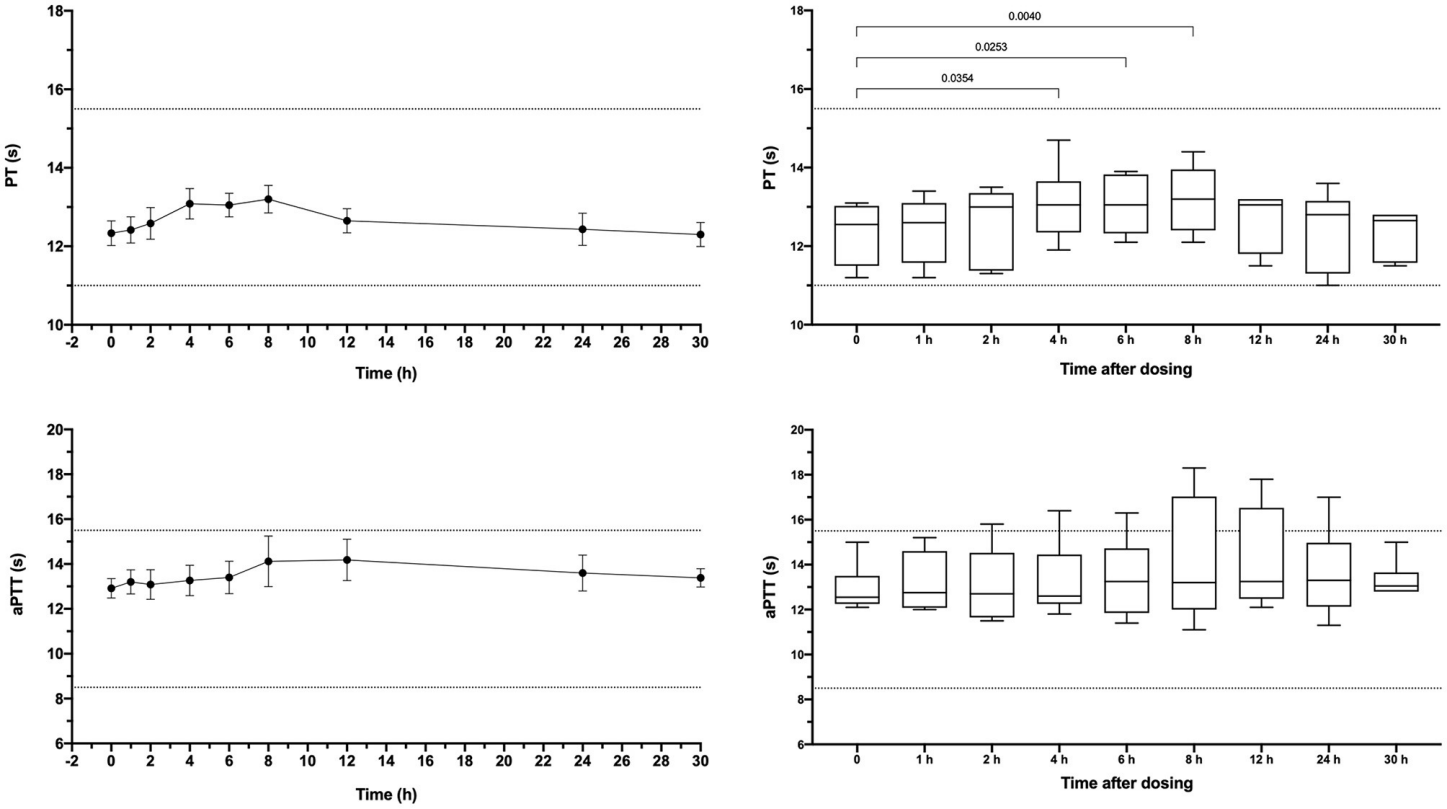
Dotplots and box-whisker plots representing the prothrombin time (PT) and activated partial thromboplastin time (aPTT) values following oral administration of apixaban (0.2 mg/kg). Data presented on the left panels represent mean ± standard deviation values, while data on the right panels represent median (middle line), 25–75% percentile (box) and min-max (whiskers) for each time point. Dotted horizontal lines represent the upper and lower bounds of the laboratory reference interval. For the box-whisker plots, comparisons between baseline and subsequent time points were conducted using the Friedman test with Dunn's *post-hoc* correction for multiple comparisons. Only those comparisons that attained *P* < 0.05 are displayed.

Dogs received a single oral dose of 2-3 mg based on bodyweight, equivalent to a mean of 0.20 ± 0.01 mg/kg. The resulting mean maximum observed plasma concentration (C_Max_) was 176 ± 126 ng/ml, which occurred at 6 h post-dosing and corresponded to an aXa of 128 ng/ml (Figure 5). Apixaban plasma concentrations and aXa activity were positively correlated R^2^ 0.733, *P* < 0.0001 (Supplementary Figure 3). Residual plasma concentrations were quantifiable at the 24-h sampling time point and declined to below the level of quantitation by the 30-h time point. Mean apixaban aXa activity remained below values typically observed for human moderate to high dose anticoagulant effect (40). Pharmacokinetic parameters following oral dosing are summarized in Table 3. It was not possible to fit a pharmacokinetic model for one dog (#6), because the concentrations continued to increase at the last sample point. The mean bioavailability of apixaban in these dogs was 28.4%, with a mean lag time (T_Lag_) of 2 h and a mean time to C_Max_ of 5 h. Oral apixaban significantly prolonged the PT at 4, 6, and 8 h compared to baseline values (Figure 6), but no PT value exceeded the upper bound of the reference interval at any time point. Oral apixaban did not have a significant effect on aPTT, and no time exceeded the upper bound of the reference interval. Tissue-factor-activated TEG tracings were not consistently affected by oral apixaban. Only R-time at the 12-h timepoint was significantly different from baseline (Supplementary Figure 4).

**Table 3:** Summary of pharmacokinetic parameters following oral administration of apixaban, mean dose 0.2 mg/kg.

**Units**	**Parameter**	**Dog 1**	**Dog 2**	**Dog 3**	**Dog 4**	**Dog 5**	**Mean**	**Std. Dev**.	**Geo Mean**	**CV%**
AUC	h*ng/ml	2,082.4	2,396.3	1,335.5	863.4	3,051.2	1,945.7	864.2	1,773.7	44.4
CL/F	ml/h/kg	98.4	79.7	155.8	220.1	68.8	124.6	63.0	113.1	50.6
C_Max_	ng/ml	236.2	294.3	165.1	71.4	348.5	223.1	108.8	195.5	48.8
K01	1/h	0.7	0.5	0.3	0.2	0.6	0.5	0.2	0.4	41.8
K01 T½	h	1.0	1.4	2.1	3.1	1.1	1.7	0.9	1.6	50.8
K10	1/h	0.2	0.2	0.3	0.2	0.2	0.2	0.1	0.2	25.9
K10 T½	h	3.8	2.8	2.1	3.1	3.6	3.1	0.7	3.0	22.4
T_Lag_	h	0.9	1.6	1.9	1.8	3.4	2.0	0.9	1.8	47.3
T_Max_	h	3.5	4.4	4.9	6.3	6.1	5.1	1.2	5.0	23.1
V/F	ml/kg	540.7	326.7	465.9	974.7	361.2	533.8	260.6	492.5	48.8
F%	%	35.0	51.5	15.0	10.0	30.4	28.4	16.6	24.1	58.5

*n = 5; data from dog 6 could not be fit with an oral model. AUC, area-under-the-curve; CL/F, systemic clearance per fraction absorbed; C_Max_, peak concentration; K01, first order absorption rate and respective half-life (T½); K10, first order elimination rate, and respective half-life (T½); V/F, volume of distribution per fraction absorbed; T_Lag_, lag time for capsule dissolution and stomach emptying; T_Max_, peak concentration; F%, fraction of oral dose absorbed*.

## Discussion

This study describes the PK and biologic activity profiles of apixaban following IV and PO administration of 0.2 mg/kg apixaban to six healthy, middle-aged mixed-breed dogs. The population used in this study was intended to be more representative of potential target patient populations than laboratory beagles (41). Intravenous administration produced high plasma apixaban concentrations and FXa inhibitory activity and caused corresponding prolongations of coagulation times and hypocoagulability per TEG traces. Apixaban, however, had limited bioavailability in dogs compared with humans. The drug was highly protein bound, with a corresponding low apparent volume of distribution typical of highly protein bound drugs (177 ml/kg). The PK of apixaban administered intravenously was highly reproducible among dogs. There was an excellent positive correlation between apixaban's measured plasma concentrations and aXa activity, such that intravenous apixaban's biologic activity was also highly reproducible. Although the aXa assay reports a value in ng/ml, this is not providing an estimate of the plasma concentration of apixaban, but rather the aXa activity of an equivalent concentration of apixaban in the assay itself. The strong, direct correlation between these two parameters suggests that measurement of apixaban plasma concentrations by LC-MS is unnecessary for routine clinical monitoring of apixaban. The apixaban aXa assay is more widely available at coagulation laboratories and is less expensive and time-consuming than LC-MS analyses.

It is noteworthy that the relationship between the plasma apixaban concentration quantitated by LC/MS and the corresponding aXa activity was non-linear following IV administration of apixaban. This is consistent with the non-linear relationship apparent following rivaroxaban administration to cats (42). This non-linear relationship is the cause of the numerical discrepancy between the values produced by the two assays that both report in ng/ml. The aXa activity assay is linear between 23-500 ng/ml. Very high plasma concentrations produce a ceiling effect where further increases in plasma concentration correspond with smaller increases in aXa activity. This is likely due to excess apixaban in the assay at very high concentrations meaning that all of the FXa in the assay is inhibited, limiting the resultant chromogenic substrate cleavage. Dilution studies could be performed to linearize the relationship, but this may not be necessary for clinical practice. We suggest that clinicians interpret very high aXa activities as indicative of overdosage and consider dose reduction or extension of the dosing interval. Repeat aXa activity measurement following dose reduction would be prudent.

In this study, we did not attempt to determine a dose that would achieve a specific therapeutic aXa activity. Attempts have been made to extrapolate a dose of rivaroxaban from humans to dogs (17), but this approach is potentially problematic because of issues related to relative potency (43). The dose used in this study was extrapolated from previous studies in cats and horses. In humans, a therapeutic aXa activity reference range for apixaban has not been established. Rather peak and trough levels observed in clinical trials using different dosing regimens are available and these values are likely indication specific (44). As such, while we can compare the aXa activities achieved in the present study with those achieved in human clinical trials, we cannot conclude whether such aXa activities would be protective in dogs at risk of thrombosis or therapeutic in dogs with existing thrombi. Future dose ranging studies and clinical trials in at-risk dog populations will be necessary to determine if achievable plasma concentrations are safe and effective. It should be noted that the apixaban calibrated aXa activity assay reports values in ng/ml. This is in contrast to aXa activities for the heparins which are reported in U/ml. It is crucial that if apixaban is to be monitored using an aXa activity assay that this assay should be calibrated using apixaban in order that the data reported here and elsewhere (25, 26) can be correctly interpreted. In humans, apixaban is typically administered at a fixed dose per indication and therapeutic targets for aXa activity have not been formally established. The range of apixaban aXa activities that result from these fixed doses have been reported (44), but these aren't true targets. Until veterinary clinical trials establish what apixaban aXa activities correspond to clinical efficacy (thromboprophylaxis without bleeding), using doses that achieve similar aXa activities to those reported in people is reasonable.

Although controlled, treatment trials of DOAC's in veterinary patients are lacking, several preclinical studies of apixaban have been performed in dogs. An *in vitro* study of dog plasma fortified with apixaban determined the inhibitor constant (Ki) of the compound for dog factor Xa 0.78 ng/ml (1.7 nM), and the concentration required to double the prothrombin time 3,079 ng/ml (6.7 μM) (28). In the study by Wong et al., the Ki value was established *in vitro* using isolated factor Xa. Consequently, the concentrations of apixaban required to inhibit the enzyme in that system were much lower than the EC50 value in our study. In contrast, the concentration required to double the PT in Wong et al. was very comparable to those generated in the present study. An estimate of the concentration required to double the PT from baseline based on the regression equation line (Figure 2B) from our study is 2,485 ng/ml. This suggests our data are very consistent with the inhibition of canine FXa demonstrated by Wong et al. A second study of the metabolism of apixaban in dogs administered 5 mg/kg of ^14^C labeled apixaban orally to male beagles and demonstrated that most of the drug circulated as apixaban, with clearance mediated by oxidative metabolism as well as excretion of the unchanged compound *via* renal (7%) and gastrointestinal routes (59%) (29). A third study from 2011 assessed the PK of apixaban in rats, dogs, and chimpanzees (30). In the study by He et al. (30), dogs were administered 0.5 mg/kg IV or PO with maximal plasma concentrations achieved after 2.8 h. The half-life of apixaban in that study was 3.2 h after intravenous administration and 4.3 h after oral administration. The oral bioavailability was reportedly 80% in that study, considerably higher than the 28.4% reported here. Unfortunately, that study did not provide details on the signalment of the dogs involved and only limited information about the oral formulation, which makes direct comparisons with our study difficult. The study by He et al. used a liquid formulation for oral administration containing methylcellulose and polysorbate 80, in contrast to the powder containing capsules used here. The liquid formulation used by He et al. would likely maximize oral bioavailability, but it is not practicable for administration to clinical patients. The differences in half-lives between the study of He et al. and ours are small but may have resulted from differences in drug formulation and the use of different breeds and ages of dogs. In the study by He et al., apixaban prolonged the factor Xa-dependent clotting time test (HEPTEST), but not the standard PT. This difference with our findings of apixaban-related prolonged PT might be explained by the use of different PT reagents between studies (Thromboplastin C-Plus, Dade Behring vs. Thromboplastin LI). Apixaban was also reported to be over 90% protein-bound (30).

A pharmacokinetic and pharmacodynamic study of apixaban in cats (26) found that the drug was more bioavailable (85.5%) in cats than was found for dogs in the present study. However, substantial interindividual variability following oral administration limited PK model fitting in cats. In that study, the half-life of oral apixaban was 3.3 h, while the half-life of intravenously administered apixaban was only 68 min. Following oral administration, peak aXa activity in cats occurred at 4 h post-administration, suggesting more rapid uptake of apixaban in cats compared to dogs in the present study, where the peak occurred at approximately 5 h. In cats, as in dogs, plasma apixaban concentrations were more highly correlated with aXa activity after IV than administration compared to oral administration. This might be due to lower aXa activity assay precision at the lower apixaban concentrations achieved after oral administration. The correlation after oral administration in the present study (R^2^ 0.733) was superior to that reported by Myers et al. for cats (−0.548) (26).

Unlike dogs and cats, PK studies of apixaban in horses have not shown a potential for clinical use. Intravenous apixaban in horses resulted in plasma concentrations that correlated with aXa activity (25), but oral administration did not lead to quantifiable apixaban concentrations in plasma. The half-life of apixaban in horses is also very short at 1.3 h, such that after a single injection, concentrations declined below the lower limit of quantitation by 8 h. In contrast, the half-life in dogs after intravenous injection is ~4.1 h, and 3.1 h after oral administration. In horses, as in dogs, the drug is highly protein bound.

In humans, the oral direct factor Xa inhibitors show dose-dependent, predictable effects that obviate the need for routine monitoring. The variation in apixaban bioavailability between a preclinical dog study (30) and our finding of relatively low bioavailability, suggests that aXa activity monitoring might be warranted to gauge anticoagulant intensity in treated dogs. We recently conducted a retrospective study of sick dogs administered rivaroxaban and monitored with a rivaroxaban calibrated aXa activity assay (45). In that population of 19 dogs, post-treatment monitoring revealed that 8/19 dogs had aXa activities below the range associated with positive clinical outcomes in humans, while an additional 8/19 dogs exceeded that range. In this group of dogs, the initial rivaroxaban dose administered was not significantly related to measured aXa activity values. Potential explanations for this lack of association include variable drug absorption, altered volumes of distribution, differences in protein binding, and inhibition of metabolism related to underlying disease conditions. These observations suggest that the consistent dose-response relationships seen in humans taking the direct Xa inhibitors might not occur in dogs, thus therapeutic drug monitoring of this class of drugs in dogs might be useful, especially in dose-finding treatment trials.

Limitations of this study include use of a single dose (0.2 mg/kg) extrapolated from previous pharmacokinetic studies of apixaban in cats and horses. The IV formulation we used was prepared as previously published (25), and we verified the final concentration of apixaban in these preparations by LC/MS. The formulation did contain excipients (dimethylacetamide, propylene glycol, and dimethyl sulfoxide) to aid drug extraction and solubilization, and it is possible that these compounds might have impacted our biologic activity assessments by interfering with blood coagulation. However, the final blood concentrations of these excipients even immediately after injection would have been low due to the small volume of injectate and the substantial dilution in the blood volume.

Use of a single oral dose precluded us from comparing anticoagulant intensity across a range of doses that might be considered in clinical patients. The lower oral bioavailability in our study compared to previous reports might have been responsible for the failure of the dose chosen to reach aXa activity values of approximately 150 ng/ml typically observed in human treatment trials (46). If the drug had been as bioavailable as previously reported, then aXa activity might have exceeded this value. The cause of the limited bioavailability documented in our study is uncertain. To ensure consistent dosing, we compounded a commercial oral formulation into custom capsules. This might have impacted the absorption of the drug, however, because the capsule might have interfered with drug dissolution (47, 48). An alternative would have been to use commercially available 2.5 mg apixaban tablets. However, this would have resulted in a much wider range of doses (0.15–0.24 mg/kg) than we achieved (0.20 ± 0.01 mg/kg). The custom compounding was performed by a licensed compounding pharmacy, but we did not confirm that the capsules contained the specified amount of drug. Capsules were stored for a maximum of 76 days prior to administration. The compounding pharmacy indicated that storage of up to 180 days was acceptable prior to usage, but we did not confirm that the apixaban was stable in this new formulation. Hence, it is possible that our use of custom capsules introduced unquantified variation into our results including under- and overdosage, impaired drug absorption and drug degradation. Future studies, including dose ranging or multidose studies should consider using the commercial tablet formulations to confirm our results and to determine if the oral bioavailability of apixaban is adequate for therapeutic usage. All dogs in the present study were fasted (as in prior reports), but it is uncertain what impact concurrent feeding might have on bioavailability.

We also only evaluated a single intravenous administration and a single oral administration. Multidose studies will be necessary to determine if steady state plasma concentrations can be effectively and safely reached and to determine the effects of chronic administration. Our study involved mixed-breed dogs to better simulate the variety of breeds of client-owned dogs receiving anticoagulants. However, it is likely that critical illness will impact some of the PK parameters by altering volumes of distribution, oral absorption, and drug metabolism. Population PK studies in the target population will be necessary to determine if the PK of apixaban in clinical practice matches that determined here. In our study, the determination of protein binding was conducted using a single concentration of apixaban generated using the IV formulation. While six biological replicates were conducted, only a single analytical replicate was performed for each dog. The variation in these biological replicates was small, but we might have determined different degrees of protein binding at different concentrations. We cannot exclude the possibility of interference in the protein binding experiments from excipients in the IV formulation, but the concentrations of these compounds were <0.02% v/v in the final samples.

The PK of apixaban in horses and in cats was best described by 2-compartment models (25, 26). In the present study, the PK data following intravenous administration for 4 of the dogs best fit a 1-compartment model, while for 2 dogs a 2-compartment model was superior, although there was little difference between the two fits for these 2 dogs. Using the “principle of parsimony” we decided to report the 1-compartment model fit for all dogs. A 1-compartment model may be the best fit for apixaban in the present study because it demonstrated very high protein binding and a low volume of distribution, hence distribution to other tissues is low. It is unclear if the difference in model preference between our study and that described for horses and cats is an interspecies difference or a methodological limitation in our study and semi-log plot analysis (Figure 1) suggests some redistribution did occur. Our ability to detect a 2nd or a 3rd compartment may have been limited by the blood sampling scheme we used, that may have missed part of the redistribution phase. In our analysis we tried different models, with different parameterization, weighting, and error structure to find the best model that fit the data using the Akaike information criterion and visual inspection of predicted vs. observed plots, and residual plots. Future studies of apixaban PK in dogs and other species may need to carefully select sampling timepoints to maximize the opportunity to identify multiple compartments.

We conducted a variety of coagulation assays with different endpoints to assess the biologic activity of apixaban in dogs and to evaluate the potential use of these tests for clinical monitoring. While these assays provide pharmacodynamic information on apixaban's anticoagulant action, they are not suited to gauge clinical efficacy for thromboprophylaxis or as treatment for dogs with overt thrombosis. Similar to other investigations of DOACs in dogs (17, 49, 50), we used TEG to assess the effect of apixaban on global hemostasis. We used a 1:50,000 TF dilution for assay activation in order to produce a sensitive assay. This might have increased the potential for influence by pre-analytical factors. Others have used higher TF concentrations including 1:100 (17, 50) and 1:3,700 (49). The PROVETS guidelines (36) recommend using both a 1:3,600 and a 1:50,000 TF concentration for small animals. This was not feasible for this study because of the high frequency of analysis and the limited number of TEG channels available. We therefore chose to use the more sensitive assay. If TEG is to be used for therapeutic monitoring or in future investigations, then standardization of the TF concentrations would be of value to enable comparisons between centers and studies. We also eliminated the hold time to facilitate sample throughput. This might have led to more hypocoagulable results, but prior studies suggest the influence of the hold time is minimal when an agonist like TF is used (35). Future studies using model systems, or at-risk clinical patients will be necessary to further define the therapeutic potential of apixaban for naturally occurring thromboembolic disease in dogs.

In summary, the present study documented the PK and biologic effects of intravenous and oral apixaban (0.2 mg/kg) in mixed-breed dogs. Intravenous apixaban had reproducible PK and demonstrated consistent concentration-dependent inhibition of factor Xa. Oral apixaban in the dogs studied here had limited bioavailability and the resulting plasma concentrations and biologic effects were variable. Consequently, drug monitoring with an apixaban-calibrated anti-Xa activity assay might be useful to assess inter-individual biologic effects. The prothrombin time can also be used to assess the anticoagulant action of the drug in dogs, but prolongation of the prothrombin time might indicate circulating concentrations higher than those associated with therapeutic dosing of apixaban in humans. Further studies will be needed to determine pharmacokinetics and pharmacodynamics after multidose administration, effects of comorbidities on pharmacokinetics and pharmacodynamics, and to develop dosing recommendations for safe and effective use of apixaban for the treatment and prevention of thromboembolic disease in dogs.

## Data Availability Statement

The original contributions presented in the study are included in the article/Supplementary Material, further inquiries can be directed to the corresponding author/s.

## Ethics Statement

The animal study was reviewed and approved by Cornell University College of Veterinary Medicine IACUC.

## Author Contributions

NH collected and analyzed data and co-wrote the manuscript. RG conceived the study, collected and analyzed data, and co-wrote the manuscript. MW, IB, and MB collected and analyzed data and edited the manuscript. MP analyzed data and edited the manuscript. All authors contributed to the article and approved the submitted version.

## Conflict of Interest

The authors declare that the research was conducted in the absence of any commercial or financial relationships that could be construed as a potential conflict of interest.

## Supplementary Material

Supplementary Figure 1 Box-whisker plots representing the only complete blood count and serum chemistry values that changed significantly between baseline and the +24 h time point following intravenous (IV) or oral (PO) administration of apixaban (0.2 mg/kg). All comparisons were performed using Wilcoxon matched-pairs signed rank tests. Data are presented as median (middle line), 25–75% percentile (box) and min-max (whiskers). Only those comparisons that attained *P* < 0.05 are displayed.

Supplementary Figure 2 Scatterplots demonstrating the correlations between apixaban plasma concentrations (abscissa) and values from tissue-factor activated thromboelastography tracings following intravenous administration of apixaban (0.2 mg/kg). Values represent the reaction time (R-time), clot formation time (K-time), clot formation angle (alpha), and maximum amplitude (MA). Solid blue lines represent the Pearson least-squares linear regression lines, while the dotted blue lines represent the corresponding 95% confidence intervals. Relevant R^2^ and associated *P*-values are displayed on each panel.

Supplementary Figure 3 Scatterplot demonstrating the positive correlation between apixaban plasma concentrations (abscissa) and the anti-Xa activity (aXa) (ordinate) following oral administration of apixaban (0.2 mg/kg). These parameters were both positively correlated with the plasma apixaban concentrations (PT R^2^ 0.599, *P* < 0.0001; aPTT R^2^ 0.430, *P* < 0.0001). The solid blue line represents the Pearson least-squares linear regression line, while the dotted blue lines represent the corresponding 95% confidence intervals.

Supplementary Figure 4 Box-whisker plots representing the reaction time (R-time), clot formation time (K-time), clot formation angle (alpha), and maximum amplitude (MA) from tissue-factor activated thromboelastography tracings. Partial thromboplastin time (aPTT) values following oral administration of apixaban (0.2 mg/kg). Data are presented as median (middle line), 25–75% percentile (box) and min-max (whiskers) for each time point. Comparisons between baseline and subsequent time points were conducted using the Friedman test with Dunn's *post-hoc* correction for multiple comparisons. Only those comparisons that attained *P* < 0.05 are displayed.

## References

[1] Johnson LR Lappin MR Baker DC. Pulmonary thromboembolism in 29 dogs: 1985-1995. J Vet Intern Med. (1999) 13:338–45. 10.1111/j.1939-1676.1999.tb02192.x 10449226

[2] Winter RL Sedacca CD Adams A Orton EC. Aortic thrombosis in dogs: presentation, therapy, and outcome in 26 cases. J Vet Cardiol. (2012) 14:333–42. 10.1016/j.jvc.2012.02.008 22591640

[3] Van Winkle TJ Bruce E. Thrombosis of the portal vein in eleven dogs. Vet Pathol. (1993) 30:28–35. 10.1177/030098589303000104 8442325

[4] Carr AP Panciera DL Kidd L. Prognostic factors for mortality and thromboembolism in canine immune-mediated hemolytic anemia: a retrospective study of 72 dogs. J Vet Intern Med. (2002) 16:504–9. 10.1111/j.1939-1676.2002.tb02378.x 12322697

[5] Respess M O'Toole TE Taeymans O Rogers CL Johnston A Webster CR. Portal vein thrombosis in 33 dogs: 1998–2011. J Vet Intern Med. (2012) 26:230–7. 10.1111/j.1939-1676.2012.00893.x 22369249

[6] Palmer KG King LG Van Winkle TJ. Clinical manifestations and associated disease syndromes in dogs with cranial vena cava thrombosis: 17 cases (1989–1996). J Am Vet Med Assoc. (1998) 213:220–4. 9676591

[7] Morris TA Marsh JJ Chiles PG Pedersen CA Konopka RG Gamst AC. Embolization itself stimulates thrombus propagation in pulmonary embolism. Am J Physiol Heart Circ Physiol. (2004) 287:H818–22. 10.1152/ajpheart.01197.2003 15044200

[8] Goggs R Chan DL Benigni L Hirst C Kellett-Gregory L Fuentes VL. Comparison of computed tomography pulmonary angiography and point-of-care tests for pulmonary thromboembolism diagnosis in dogs. J Small Anim Pract. (2014) 55:190–7. 10.1111/jsap.12185 24521253 PMC4477636

[9] Agnelli G Buller HR Cohen A Curto M Gallus AS Johnson M. Oral apixaban for the treatment of acute venous thromboembolism. N Engl J Med. (2013) 369:799–808. 10.1056/NEJMoa1302507 23808982

[10] Buller HR Prins MH Lensin AW Decousus H Jacobson BF Minar E. Oral rivaroxaban for the treatment of symptomatic pulmonary embolism. N Engl J Med. (2012) 366:1287–97. 10.1056/NEJMoa1113572 22449293

[11] Granger CB Alexander JH McMurray JJ Lopes RD Hylek EM Hanna M. Apixaban versus warfarin in patients with atrial fibrillation. N Engl J Med. (2011) 365:981–92. 10.1056/NEJMoa1107039 21870978

[12] Giustozzi M Franco L Vedovati MC Becattini C Agnelli G. Safety of direct oral anticoagulants versus traditional anticoagulants in venous thromboembolism. J Thromb Thrombolysis. (2019). 48:439–53. 10.1007/s11239-019-01878-x 31104194

[13] Blais MC Bianco D Goggs R Lynch AM Palmer L Ralph A. Consensus on the rational use of antithrombotics in veterinary critical care (CURATIVE): domain 3-defining antithrombotic protocols. J Vet Emerg Crit Care. (2019) 29:60–74. 10.1111/vec.12795 30654416

[14] Goggs R Blais MC Brainard BM Chan DL deLaforcade AM Rozanski E. American College of Veterinary Emergency and Critical Care (ACVECC) Consensus on the Rational Use of Antithrombotics in Veterinary Critical Care (CURATIVE) guidelines: small animal. J Vet Emerg Crit Care. (2019) 29:12–36. 10.1111/vec.12801 30654421

[15] Eymin G Jaffer AK. Thromboprophylaxis in major knee and hip replacement surgery: a review. J Thromb Thrombolysis. (2012) 34:518–25. 10.1007/s11239-012-0751-5 22653706

[16] Helmond SE Polzin DJ Armstrong PJ Finke M Smith SA. Treatment of immune-mediated hemolytic anemia with individually adjusted heparin dosing in dogs. J Vet Intern Med. (2010) 24:597–605. 10.1111/j.1939-1676.2010.0505.x 20384956

[17] Conversy B Blais MC Dunn M Gara-Boivin C Del Castillo JRE. Anticoagulant activity of oral rivaroxaban in healthy dogs. Vet J. (2017) 223:5–11. 10.1016/j.tvjl.2017.03.006 28671072

[18] Conversy B Blais MC Dunn M Gara-Boivin C Carioto L del Castillo JR. Rivaroxaban demonstrates in vitro anticoagulant effects in canine plasma. Vet J. (2013) 198:437–43. 10.1016/j.tvjl.2013.08.001 24053991

[19] Yang VK Cunningham SM Rush JE de Laforcade A. The use of rivaroxaban for the treatment of thrombotic complications in four dogs. J Vet Emerg Crit Care. (2016) 26:729–36. 10.1111/vec.12466 26990131

[20] Morassi A Bianco D Park E Nakamura RK White GA. Evaluation of the safety and tolerability of rivaroxaban in dogs with presumed primary immune-mediated hemolytic anemia. J Vet Emerg Crit Care. (2016) 26:488–94. 10.1111/vec.12480 27074368

[21] Tritschler T Castellucci LA. It's time for head-to-head trials with direct oral anticoagulants. Thromb Res. (2019) 180:64–9. 10.1016/j.thromres.2019.05.019 31226664

[22] Dawwas GK Brown J Dietrich E Park H. Effectiveness and safety of apixaban versus rivaroxaban for prevention of recurrent venous thromboembolism and adverse bleeding events in patients with venous thromboembolism: a retrospective population-based cohort analysis. Lancet Haematol. (2019) 6:e20–8. 10.1016/S2352-3026(18)30191-1 30558988

[23] Castellucci LA Cameron C Le Gal G Rodger MA Coyle D Wells PS. Clinical and safety outcomes associated with treatment of acute venous thromboembolism: a systematic review and meta-analysis. JAMA. (2014) 312:1122–35. 10.1001/jama.2014.10538 25226478

[24] Cohen AT Hamilton M Mitchell SA Phatak H Liu X Bird A. Comparison of the novel oral anticoagulants apixaban, dabigatran, edoxaban, and rivaroxaban in the initial and long-term treatment and prevention of venous thromboembolism: systematic review and network meta-analysis. PLoS ONE. (2015) 10:e0144856. 10.1371/journal.pone.0144856 26716830 PMC4696796

[25] Serpa PBS Brooks MB Divers T Ness S Birschmann I Papich MG. Pharmacokinetics and pharmacodynamics of an oral formulation of apixaban in horses after oral and intravenous administration. Front Vet Sci. (2018) 5:304. 10.3389/fvets.2018.00304 30564584 PMC6288471

[26] Myers JA Wittenburg LA Olver CS Martinez CM Bright JM. Pharmacokinetics and pharmacodynamics of the factor Xa inhibitor apixaban after oral and intravenous administration to cats. Am J Vet Res. (2015) 76:732–8. 10.2460/ajvr.76.8.732 26207972

[27] Borgarelli M Lanz O Pavlisko N Abbott JA Menciotti G Aherne M. Mitral valve repair in dogs using an ePTFE chordal implantation device: a pilot study. J Vet Cardiol. (2017). 19:256–67. 10.1016/j.jvc.2017.03.002 28576476

[28] Wong PC Crain EJ Xin B Wexler RR Lam PY Pinto DJ. Apixaban, an oral, direct and highly selective factor Xa inhibitor: in vitro, antithrombotic and antihemostatic studies. J Thromb Haemost. (2008) 6:820–9. 10.1111/j.1538-7836.2008.02939.x 18315548

[29] Zhang D He K Raghavan N Wang L Mitroka J Maxwell BD. Comparative metabolism of 14C-labeled apixaban in mice, rats, rabbits, dogs, and humans. Drug Metab Dispos. (2009) 37:1738–48. 10.1124/dmd.108.025981 19420130

[30] He K Luettgen JM Zhang D He B Grace JE Jr Xin B. Preclinical pharmacokinetics and pharmacodynamics of apixaban, a potent and selective factor Xa inhibitor. Eur J Drug Metab Pharmacokinet. (2011) 36:129–39. 10.1007/s13318-011-0037-x 21461793

[31] Lunsford KV Mackin AJ Langston VC Brooks M. Pharmacokinetics of subcutaneous low molecular weight heparin (enoxaparin) in dogs. J Am Anim Hosp Assoc. (2009) 45:261–7. 10.5326/0450261 19887383

[32] Pouzot-Nevoret C Barthelemy A Cluzel M Verwaerde P Bonnet-Garin JM Goy-Thollot I. Enoxaparin has no significant anticoagulation activity in healthy Beagles at a dose of 0.8 mg/kg four times daily. Vet J. (2016) 210:98–100. 10.1016/j.tvjl.2016.01.018 26896297

[33] McMillan CJ Livingston A Clark CR Dowling PM Taylor SM Duke T. Pharmacokinetics of intravenous tramadol in dogs. Can J Vet Res. (2008) 72:325–31. 18783021 PMC2442675

[34] KuKanich B Papich MG. Pharmacokinetics of tramadol and the metabolite O-desmethyltramadol in dogs. J Vet Pharmacol Ther. (2004) 27:239–46. 10.1111/j.1365-2885.2004.00578.x 15305853

[35] Wiinberg B Jensen AL Rojkjaer R Johansson P Kjelgaard-Hansen M Kristensen AT. Validation of human recombinant tissue factor-activated thromboelastography on citrated whole blood from clinically healthy dogs. Vet Clin Pathol. (2005) 34:389–93. 10.1111/j.1939-165X.2005.tb00066.x 16270265

[36] Goggs R Brainard B de Laforcade AM Flatland B Hanel R McMichael M. Partnership on Rotational ViscoElastic Test Standardization (PROVETS): evidence-based guidelines on rotational viscoelastic assays in veterinary medicine. J Vet Emerg Crit Care. (2014) 24:1–22. 10.1111/vec.12144 24422679

[37] Kuhn J Gripp T Flieder T Hammerschmidt A Hendig D Faust I. Measurement of apixaban, dabigatran, edoxaban and rivaroxaban in human plasma using automated online solid-phase extraction combined with ultra-performance liquid chromatography-tandem mass spectrometry and its comparison with coagulation assays. Clin Chim Acta. (2018) 486:347–56. 10.1016/j.cca.2018.08.017 30114406

[38] Yamaoka K Nakagawa T Uno T. Application of Akaike's information criterion (AIC) in the evaluation of linear pharmacokinetic equations. J Pharmacokinet Biopharm. (1978) 6:165–75. 10.1007/BF01117450 671222

[39] Avezum A Lopes RD Schulte PJ Lanas F Gersh BJ Hanna M. Apixaban in comparison with warfarin in patients with atrial fibrillation and valvular heart disease: findings from the apixaban for reduction in stroke and other thromboembolic events in atrial fibrillation (ARISTOTLE) trial. Circulation. (2015) 132:624–32. 10.1161/CIRCULATIONAHA.114.014807 26106009

[40] Shin H Cho MC Kim RB Kim CH Choi NC Kim SK. Laboratory measurement of apixaban using anti-factor Xa assays in acute ischemic stroke patients with non-valvular atrial fibrillation. J Thromb Thrombolysis. (2018) 45:250–6. 10.1007/s11239-017-1590-1 29198080

[41] Fleischer S Sharkey M Mealey K Ostrander EA Martinez M. Pharmacogenetic and metabolic differences between dog breeds: their impact on canine medicine and the use of the dog as a preclinical animal model. AAPS J. (2008) 10:110–9. 10.1208/s12248-008-9011-1 18446511 PMC2747081

[42] Dixon-Jimenez AC Brainard BM Brooks MB Nie B Arnold RD Loper D. Pharmacokinetic and pharmacodynamic evaluation of oral rivaroxaban in healthy adult cats. J Vet Emerg Crit Care. (2016) 26:619–29. 10.1111/vec.12524 27599304

[43] Conversy B Blais MC Dunn M Gara-Boivin C Del Castillo JRE. Corrigendum to Anticoagulant activity of oral rivaroxaban in healthy dogs [Vet. J. 223 (2017) 5-11]. Vet J. (2020) 263:105522. 10.1016/j.tvjl.2020.105522 32928491

[44] Byon W Garonzik S Boyd RA Frost CE. Apixaban: a clinical pharmacokinetic and pharmacodynamic review. Clin Pharmacokinet. (2019) 58:1265–79. 10.1007/s40262-019-00775-z 31089975 PMC6769096

[45] Tracy AL Goggs R Brooks MB Lynch AM. Clinical features and post treatment monitoring of 19 dogs administered rivaroxaban (2018–2020). J Vet Emerg Crit Care. (in press). 10.1111/vec.13199 35442563

[46] Cuker A Siegal DM Crowther MA Garcia DA. Laboratory measurement of the anticoagulant activity of the non-vitamin K oral anticoagulants. J Am Coll Cardiol. (2014) 64:1128–39. 10.1016/j.jacc.2014.05.065 25212648 PMC4167772

[47] Melia CD Davis SS. Review article: mechanisms of drug release from tablets and capsules. I: disintegration. Aliment Pharmacol Ther. (1989) 3:223–32. 10.1111/j.1365-2036.1989.tb00208.x 2520618

[48] Melia CD Davis SS. Review article: mechanisms of drug release from tablets and capsules. 2. dissolution. Aliment Pharmacol Ther. (1989) 3:513–25. 10.1111/j.1365-2036.1989.tb00243.x 2518865

[49] Bae J Kim H Kim W Kim S Park J Jung DI. Therapeutic monitoring of rivaroxaban in dogs using thromboelastography and prothrombin time. J Vet Intern Med. (2019) 33:1322–30. 10.1111/jvim.15478 30859645 PMC6524124

[50] Lynch AM Ruterbories LK Griffith EH Hanel RM Stablein AP Brooks MB. Evaluation of point-of-care coagulation tests as alternatives to anti-Xa activity for monitoring the anticoagulant effects of rivaroxaban in healthy dogs. J Vet Emerg Crit Care. (2021) 31:18–24. 10.1111/vec.13011 33118685

