# Supplementary figures and images for: Pharmacokinetics and Biologic Activity of Apixaban in Healthy Dogs
Source: Front Vet Sci. 2021 Jul 5;8:702821. doi: 10.3389/fvets.2021.702821 (PMC8287028; doi:10.3389/fvets.2021.702821)

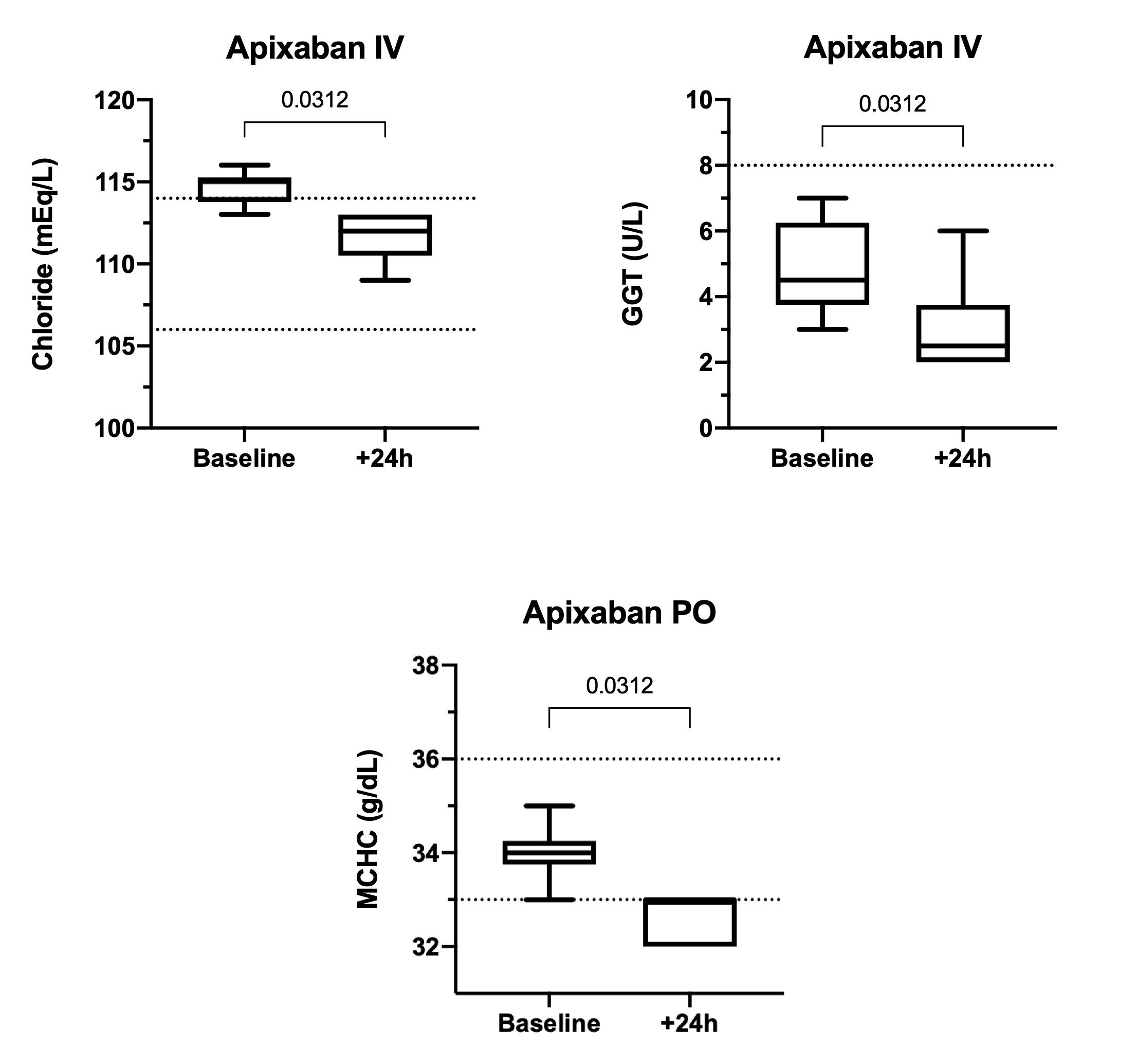

Supplement: Supplementary Figure 1 — Box-whisker plots representing the only complete blood count and serum chemistry values that changed significantly between baseline and the +24 h time point following intravenous (IV) or oral (PO) administration of apixaban (0.2 mg/kg). All comparisons were performed using Wilcoxon matched-pairs signed rank tests. Data are presented as median (middle line), 25–75% percentile (box) and min-max (whiskers). Only those comparisons that attained P < 0.05 are displayed. [file Image_1.TIFF]

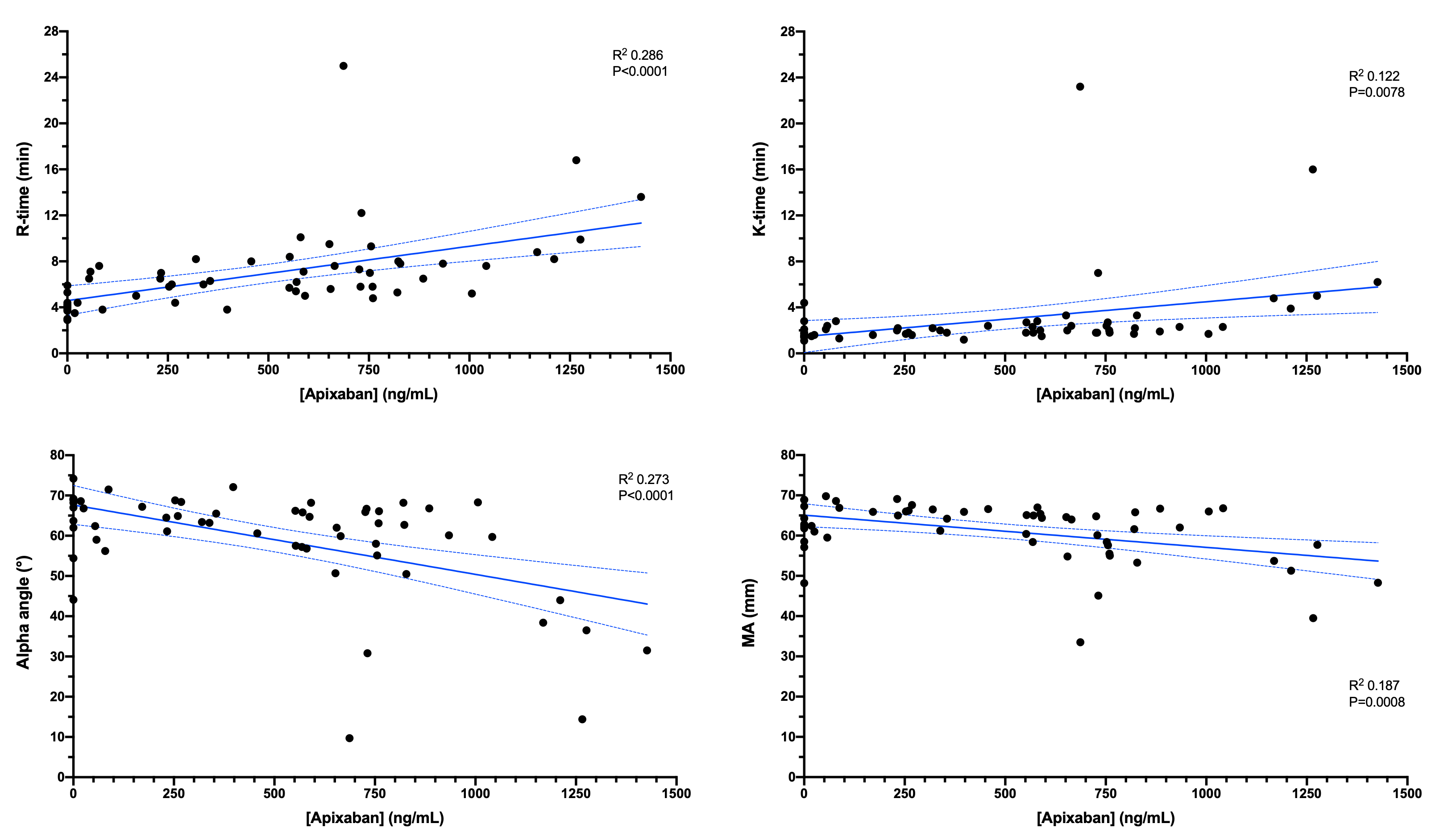

Supplement: Supplementary Figure 2 — Scatterplots demonstrating the correlations between apixaban plasma concentrations (abscissa) and values from tissue-factor activated thromboelastography tracings following intravenous administration of apixaban (0.2 mg/kg). Values represent the reaction time (R-time), clot formation time (K-time), clot formation angle (alpha), and maximum amplitude (MA). Solid blue lines represent the Pearson least-squares linear regression lines, while the dotted blue lines represent the corresponding 95% confidence intervals. Relevant R2 and associated P-values are displayed on each panel. [file Image_2.TIFF]

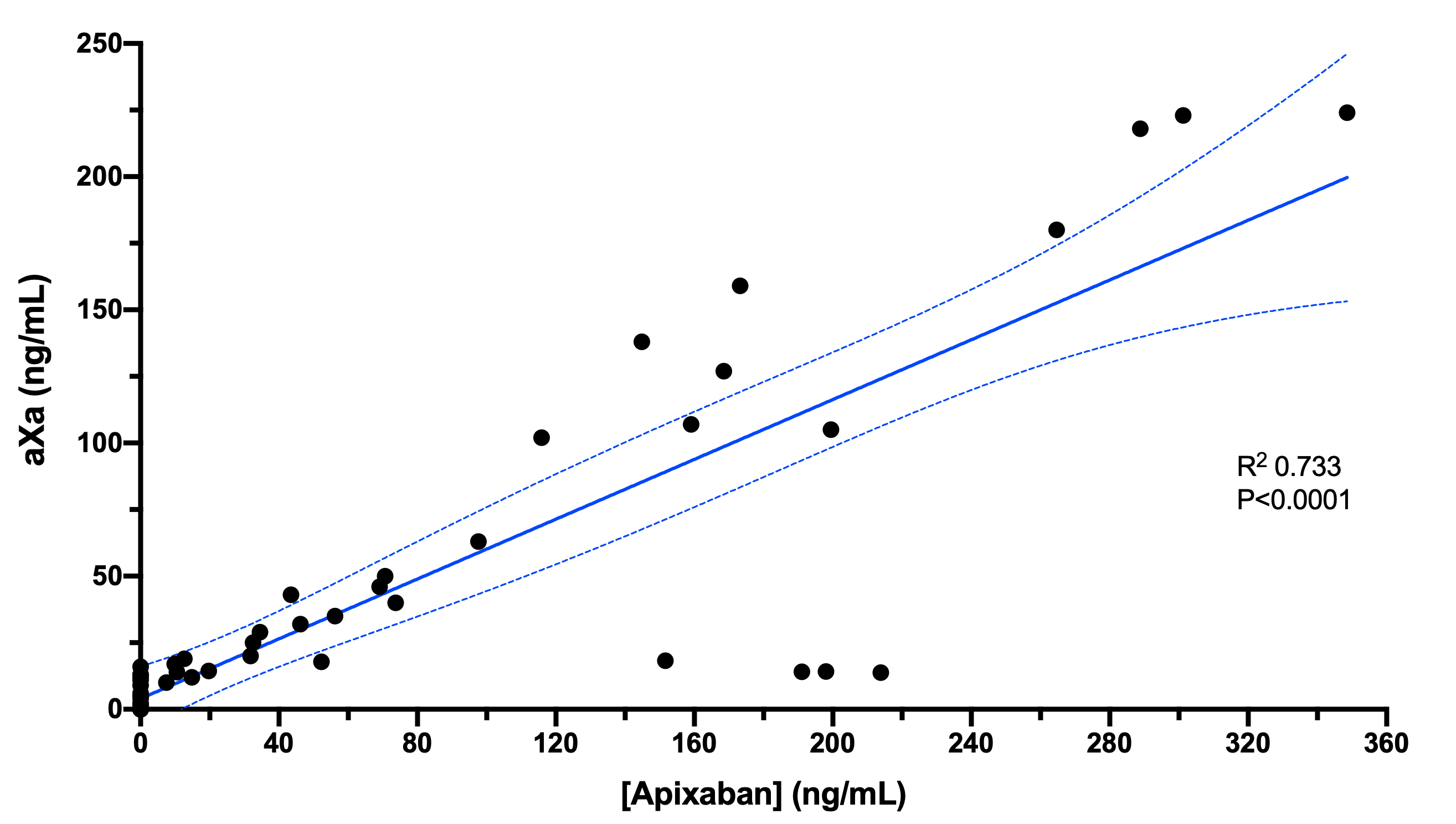

Supplement: Supplementary Figure 3 — Scatterplot demonstrating the positive correlation between apixaban plasma concentrations (abscissa) and the anti-Xa activity (aXa) (ordinate) following oral administration of apixaban (0.2 mg/kg). These parameters were both positively correlated with the plasma apixaban concentrations (PT R2 0.599, P < 0.0001; aPTT R2 0.430, P < 0.0001). The solid blue line represents the Pearson least-squares linear regression line, while the dotted blue lines represent the corresponding 95% confidence intervals. [file Image_3.TIFF]

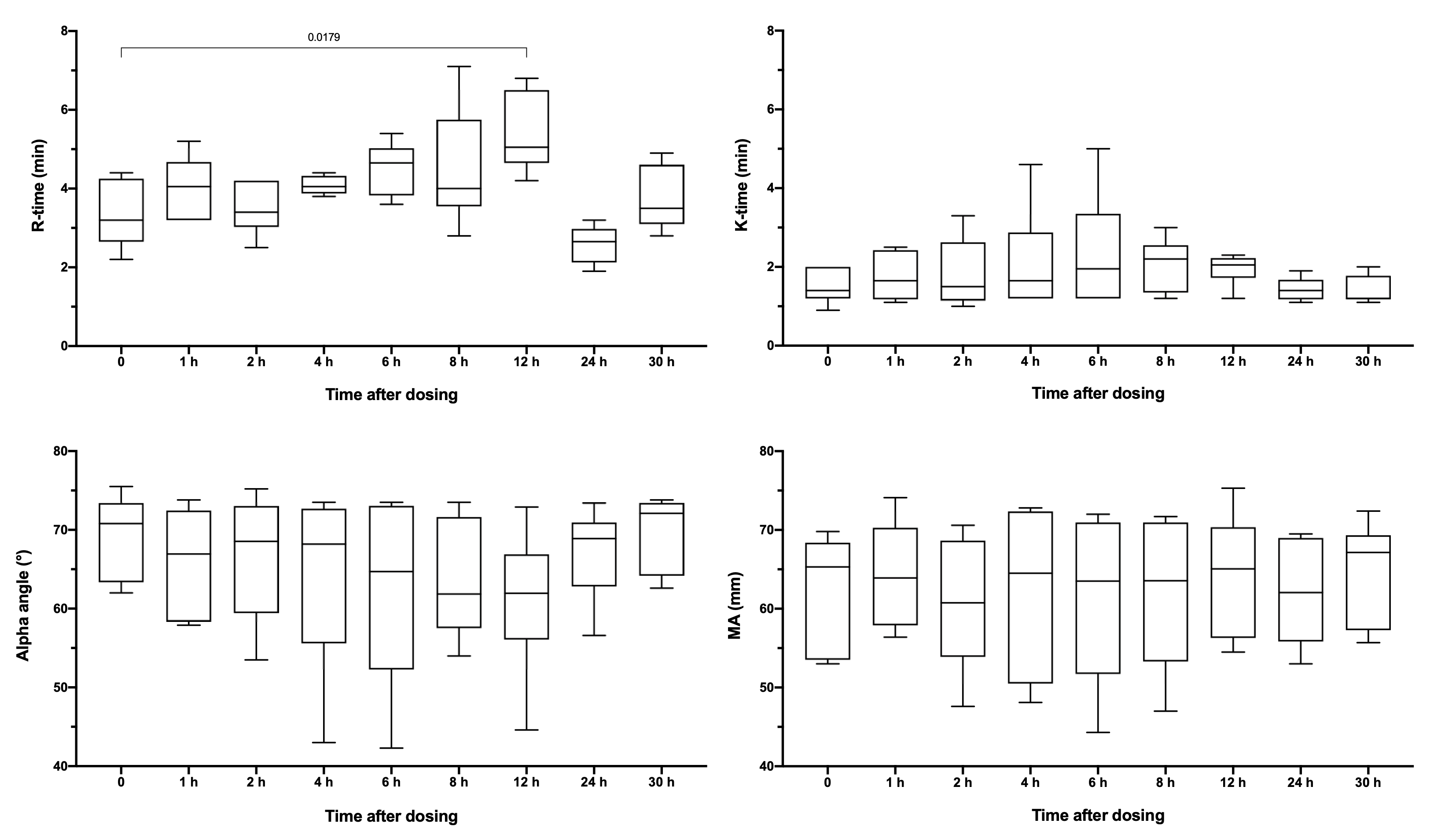

Supplement: Supplementary Figure 4 — Box-whisker plots representing the reaction time (R-time), clot formation time (K-time), clot formation angle (alpha), and maximum amplitude (MA) from tissue-factor activated thromboelastography tracings. Partial thromboplastin time (aPTT) values following oral administration of apixaban (0.2 mg/kg). Data are presented as median (middle line), 25–75% percentile (box) and min-max (whiskers) for each time point. Comparisons between baseline and subsequent time points were conducted using the Friedman test with Dunn's post-hoc correction for multiple comparisons. Only those comparisons that attained P < 0.05 are displayed. [file Image_4.TIFF]
